# Exploring University Students' Attitudes towards Primary Care: Evidence from a Cross-Sectional Study

**DOI:** 10.1155/2020/1043809

**Published:** 2020-04-16

**Authors:** Gianluca Voglino, Giuseppina Lo Moro, Maria Rosaria Gualano, Fabrizio Bert, Roberta Siliquini

**Affiliations:** Department of Public Health Sciences, University of Turin, Turin, Italy

## Abstract

The general practitioner (GP) has a significant role in primary care, being more than a gatekeeper to health services access. In Italy, if a citizen moves for studies for at least 3 months, he/she can choose temporarily another GP in the new city (the so-called “Healthcare Domicile” (HD)). The aims were to estimate the university students' knowledge about the HD, evaluate the frequency of the transition to another GP, and assess the university students' attitudes towards the primary care services. In 2018, a cross-sectional pilot study was performed in study rooms among students attending the University of Turin Participation was voluntary, anonymous, and without compensation. A 25-item questionnaire collected information about the sociodemographic characteristics, health services use, health conditions and medications, HD knowledge, and HD use. The outcomes were having the GP far away, knowing HD, and not moving the GP even if aware of HD. Chi-square test, Mann–Whitney *U*-test, and logistic regression analyses were performed. The significance level was *p* ≤ 0.05. Participants were 388 and those who knew HD were 45.36%. Among those who moved to Turin (44.85%), 77.67% knew HD but did not move the GP anyway. The 72.68% used medications without prescription (the most taken: nonsteroidal anti-inflammatory drugs and antibiotics). Age, nationality, and degree course type could be predictors for outcomes considered. HD knowledge was associated with a different use of healthcare resources. The data of the present paper suggest that further studies are required to better understand the framework connected with the university students' access to primary healthcare. Our results highlighted the need to implement campaigns targeted to university students to spread information about the HD and a more appropriate use of the healthcare services and medications.

## 1. Introduction

According to the Alma-Ata Declaration, Primary Health Care (PHC) is based on equity, participation, intersectoral action, appropriate technology, and central responsibility of the health system [1]. Primary Care (PC) is a subset of PHC and it is first-contact, accessible, continuous, comprehensive, and coordinated care. The general practitioner (GP) is the most significant player in PC since he/she is “the only clinician who operates at the nine levels of care: prevention, presymptomatic detection of disease, early diagnosis, diagnosis of established disease, management of disease, management of disease complications, rehabilitation, palliative care and counselling” [2]. Therefore, the GPs should be considered more than gatekeepers to health services access.

In Italy, PC is provided by the Local Health Authorities who are responsible, among other tasks, for coordinating the GPs activities. The GPs are self-employed and independent doctors, and their salary depends on a capitation fee on the number of registered patients, increased by a fee-for-service part for specific activities. Each GP can have a maximum of 1500 patients for which the healthcare provided by their GP is free of charge. Every citizen above the age of 18 can choose a GP who is registered in a list and who is working for the Local Health Authority where the citizen has his/her registered residence [3]. If a citizen moves for studies or work to another town (and consequently to another Local Health Authority) for at least 3 months, without changing the registered residence, he/she can choose temporarily another GP in the city where he/she moved to [4]. Such an opportunity is called “Healthcare Domicile” (HD) and it may be renewed every year [4]. In order to benefit from the HD, the citizen must go to register to the new Local Health Authority where he/she moved to. Without this registration, citizens can go to a GP outside the Local Health Authority where they have their registered residence but they have to pay for the visits [4].

This legal provision is particularly important for university students. Indeed, more than 21% of all university students in Italy moved for study purposes: the national data of 2016 reported that more than 350,000 students moved to another town to study [5].

According to these data, a remarkable number of university students might be involved in this legal provision. Although they can be considered a young and healthy population, the GP is still crucial for them, considering their role as health counsellors. Nevertheless, the use of such a service is related to the knowledge of the service itself by the population.

Therefore, the main purposes of this study were to estimate the knowledge about the HD among university students and evaluate the frequency of the transition to another GP. To our knowledge, in literature, no similar study has been performed to assess this topic. Secondly, the study aimed to assess the university students' attitudes towards the PHC services, such as the use of GPs and doctors on call (i.e., the out-of-hours service doctors when the GP service is unavailable).

## 2. Materials and Methods

### 2.1. Sample

A cross-sectional pilot study was performed between June and September 2018 among a convenience sample of students attending the “Università degli Studi di Torino” (UniTO) in Torino, Italy. STROBE guidelines for reporting observational studies were used [6]. The minimum sample size for ensuring student population representativeness in a descriptive study using random (not cluster) sampling (*n* = 263) was calculated through the software EpiInfo 7.0, based on the following data: population dimension (76700 people matriculated to UniTO in 2017/2018 [7]), expected frequency of UniTO students who moved to Torino for studying (22% [7]), acceptable margin of error (5%), and confidence level (95%).

Participation was voluntary, anonymous, and without compensation. The questionnaire was distributed in study rooms in Torino. The researchers ensured the participants' anonymity and the observance of ethical principles: prior to the survey administration, the aims of the study were explained and the participants were asked to sign an informed consent form.

### 2.2. Questionnaire

The questionnaire was composed of twenty-five items. The questions from one to eleven were about sociodemographic characteristics (e.g., gender, age, nationality, university attended, city of origin, and movements). From item twelve to item sixteen, data about the use of health services were collected (e.g., doctor on call and GP visits from the university beginning, emergency service use, and private doctors use). Through the question seventeen, it was asked about the city where the GP resided. The questions eighteen to twenty-three focused on health conditions and medications use (e.g., chronic diseases, participation in screening, and habitual medications). Finally, the last two items were on knowledge about the HD and the use of this opportunity.

### 2.3. Statistical Analysis

The results were analysed using the STATAMP14 statistical software (Stata Corp., College Station, TX, 2014). Firstly, descriptive analyses were performed with results expressed in frequencies and percentages for categorical variables, while quantitative variables were reported as the median and interquartile range (IQR), due to a nonnormal distribution. The normality of the distributions was tested by the Shapiro–Wilk test.

Three outcomes were considered:

- Having the GP far away
- Knowing about the HD
- Not moving the GP even if aware of HD

To create the first outcome, information about the city where participants lived and the city where GPs resided was matched. So, having the GP far away was compared to having the GP in the same city or in the same province. The second outcome was the answer to the 24^th^ question of the survey about the knowledge on HD, which had a dichotomous answer (“yes” or “no”). The third outcome was created excluding people who did not move to Torino, because they have been living there since before the university or because they preferred to commute, and people who did not know about HD. Then, the remaining participants, who affirmed to know about HD, were split into two groups: one with those who moved their GP to Torino and one with those who did not move their GP.

Several independent variables were used to evaluate the role of different characteristics of the participants on the abovementioned outcomes. Demographic information such as age, gender, and nationality was included. Moreover, university-related variables were considered, e.g., the year of the course at the university, the degree of the course, the kind of the course, and years passed from the transfer to Turin. Finally, variables that described the use of healthcare services and the medical needs of the students during their university path were included: having gone to a doctor privately since the beginning of the university, number of visits to a doctor on call since the beginning of the university, number of visits to the emergency room since the beginning of the university, having a chronic disease, being followed by a specialist regularly, taking medications regularly, and using medications without medical prescription.

For each outcome, chi-square analyses (or Fisher's exact tests, if any expected frequency was less/equal to 5) and Mann–Whitney *U*-tests were performed to determine differences between groups. Missing values were excluded from the abovementioned analyses. Univariate and multivariable logistic regressions were conducted to assess the potential role of sociodemographic, university-related, and health-related variables. The covariates to be included in the model were selected using a stepwise forward selection process, with a univariate *p* value <0.25 as the main criterion [8] and with age and gender as potential confounders. A two-tailed *p* value <0.05 was considered statistically significant for all analyses.

## 3. Results and Discussion

### 3.1. Results

#### 3.1.1. Characteristics of the Sample

A total of 437 questionnaires were collected. Among them, 9 were excluded for incompleteness and 40 because the participants did not attend UniTO. Therefore, 388 (88.79%) were appropriately filled and used for the analyses.

The majority of the sample was Italian (*n* = 376, 96.91%). The females were 247 (63.82%) and the median age was 23 (IQR 22–25). The majority was attending a Bachelor's degree program (*n* = 168, 43.98%), while 90 (23.56%) were attending a Master's degree program as a second-level academic qualification and 124 (32.46%) a Master's degree program as a single cycle degree course. The median year attended at the university was the second (IQR 1–3) while the median of the years passed from the transfer to Torino was 2 (IQR 2–4). Among the participants, 87 studied Medicine and related courses (22.42%), 90 Natural Sciences (23.20%), 53 Legal, Political, Economic, or Social Sciences (13.66%), 154 Humanities (39.69%), and 4 Agricultural or Veterinary courses (1.03%).

More than half of the participants stated that they have gone to a doctor privately since the beginning of the university (*n* = 234, 60.31%). Specifically, 53 participants went privately to a GP (22.08%), while 206 went to a specialist (85.83%) (it was possible to indicate more options). A total of 41 people (10.57%) declared to suffer from a chronic disease while 179 to be regularly followed by a specialist (46.13%) (e.g., ophthalmologist, gynaecologist, and allergist). Since the beginning of the university, the median number of visits to the doctor on call was 0 (IQR 0–0), to a GP 3 (IQR 1–6), and to the emergency room 0 (IQR 0–1). Only 27.94% of women stated to undergo regular cervical cancer screening. More than one-third (130, 33.51%) affirmed to take medications regularly (e.g., contraceptive pill, asthma treatment, and insulin). The majority used medications without a medical prescription (*n* = 282, 72.68%), with the following regularity: daily (4.26%), more than once a week (51.77%), more than once a month (35.46%), more than once a year (5.67%), and less than once a year (2.84%). The medications most taken without prescription were nonsteroidal anti-inflammatory drugs (NSAIDs) (63.14%), antibiotics (4.38%), and supplements or vitamins (3.35%).

The participants who knew about the HD were 176 (45.36%). The students who did not move to Torino for studying, because they have been living there since before the university or because they preferred to commute, were 214 (55.15%). The students who moved themselves and their GP to Torino were 24 (6.19%) and those who moved but did not move their GP to Torino were 150 (38.66%). Therefore, people with the GP in Torino or in the same province were 237 (61.08%) and people with the GP far away from Torino were 151 (38.92%). Among the participants who moved to Torino, 23 (22.33%) knew about HD and moved their GP and 80 (77.67%) knew such opportunity but did not move their GP.

#### 3.1.2. Having the GP Far Away

The analyses showed a significant association between having the GP far away and several variables, such as the year of the course at the university, the years passed from the transfer to Torino, the degree course attended, the kind of course attended, the number of GP visits, and the knowledge about HD (Table 1).

The multivariable logistic regression demonstrated some variables as predictors of this first outcome. In particular, the Italians and the Humanities students had a higher likelihood of having the GP far away. People who knew about HD had a lower probability instead (Table 2).

#### 3.1.3. Knowing about the Healthcare Domicile

In our sample, the chi-square tests showed a significant association between the knowledge about HD and the degree of course (*p*=0.044). When examining all combinations, it was seen that the participants who attended a Bachelor's degree program and who did not know about HD were observed significantly more than expected (*n* = 103, 49.28%, adjusted residual (AR) = 2.295). The participants who attended a Master's degree program as a second-level academic qualification and who did not know about HD were observed significantly less than expected instead (*n* = 41, 19.62%, AR = −1.996).

Not knowing about HD occurred in 164 people who used medications without prescription (77.36%, AR = 2.270) compared with 48 people who did not use such medications (22.64%, AR = −2.270) (*p*=0.023, chi-square test).

Moreover, the age was greater in the group who knew about HD (median age of 24, IQR 22–26) compared with the group who did not (23, IQR 21–25) (*p* < 0.001, Mann–Whitney *U*-test). Similarly, the number of visits to a doctor on call was higher in the group who affirmed to know (median of 0, IQR 0–0) compared with the other group (0, IQR 0–1) (*p*=0.012, Mann–Whitney *U*-test).

The multivariable logistic regression reported that the higher the age was, the more the participants were likely to know about HD. Similarly, the higher the number of visits to a doctor on call was, the more the students were prone to be aware. Instead, people who have gone to a doctor privately since the university beginning and who used to take medications without prescription had a lower probability of knowing. Also, those who attended Legal, Political, Economic, or Social Sciences courses had a lower likelihood if compared to those who attended Medicine and related courses (Table 2).

#### 3.1.4. Not Moving the GP Even If Aware of Healthcare Domicile

Significant associations were reported between the third outcome and several variables, such as the year of the course at the university, the kind of the course attended, having gone to a doctor privately since the beginning of the university, the number of visits to a doctor on call, and the number of visits to a GP (Table 3).

The participants who had gone to a doctor privately since the beginning of the university had a lower likelihood of not moving the GP even if aware of HD. The higher the years passed from the transfer to Torino were, the more the students were prone not to move the GP even if aware, while the opposite occurred for the number of visits to a doctor on call. The Humanities students had a higher risk of not moving the GP even if aware, if compared to those who attended Medicine or related courses (Table 4).

### 3.2. Discussion

#### 3.2.1. Summary

The present pilot study aimed to investigate the knowledge among university students about the chance to move the GP temporarily for study purposes (i.e., the HD) and evaluate the frequency of the transition to another GP. From our sample, it resulted that less than half of the participants were aware of this opportunity and most of the interviewed students refrained from that. The sample was mainly composed of Italian and female students. These data are consistent with the statistics from the whole students' population, confirming the representativeness of our sample. In fact, according to the official website of UniTO, where the sample was recruited, only around 6% are foreign students and 61% of the students are female [7]. The univariate analyses showed that different factors were significantly associated either with the knowledge about HD or with the use of such opportunity, as reported in tables. When the multivariable logistic regression was performed, it emerged that the age, nationality, and type of degree course attended could be considered predictors for the investigated outcomes. To our knowledge, no similar study has been performed to assess this topic and, therefore, no comparison can be made. Interestingly, the knowledge about HD was associated with a different use of healthcare resources. Additionally, these data were confirmed when the analyses were carried out by assessing a subgroup of subjects who refrained from moving the GP even if aware of HD.

#### 3.2.2. Strengths and Limitations

This study has some strengths and limitations that should be acknowledged. The main strengths are that it is one of the first studies about this topic and it reached the minimum sample size for ensuring representativeness at the local level. However, it represents a pilot study; therefore, the sample was recruited in an opportunistic way and the study design included students from one university, reducing the representativeness at the national level. It must be stated that all the efforts to minimize possible bias were made in the preanalytical and analytical phases. Additionally, these limitations could be justified considering the explorative purpose of the study. Indeed, few data are reported in the literature regarding health issues and knowledge of the students moving to another city for study purposes.

#### 3.2.3. Comparison with Existing Literature

The knowledge about HD was associated with a different use of health resources. These data could be connected with a limited use of public PHC resources. Indeed, a surprisingly high percentage of students consulted a private specialist. This could partially explain the increase in the out-of-pocket expenditure recorded in recent years. In fact, the Italian population spent in 2016 more than 5,7 billion euros for medical and paramedical outpatient services [9]. Furthermore, almost three out of four assumed medications without consulting a physician on a regular basis. Particularly, half of the subjects took drugs without consulting a physician more than once per week. Considering the type of medications taken without medical prescription, NSAIDs were the most frequent (more than 60%). These data are in line with the data shown in studies performed in other countries with the objective to evaluate the prevalence of self-medication and self-prescription [10–12]. It must be stated that NSAIDs are responsible for hospitalizations associated with the occurrence of complications related to the drugs [13], especially considering that students' knowledge about NSAIDs is low, according to a study published in 2015 [14]. In addition, the percentage of students taking antibiotics without consulting a physician raises an issue of particular concern. Nevertheless, self-medication with antibiotics resulted to be less frequent in our sample compared to other countries [15, 16]. However, the issue should not be underestimated, considering the role played by antibiotic misuse in the rise of antimicrobial resistance in the developed countries [17] and the general population and students' low awareness on this topic [18, 19].

Lastly, the subjects who did not move their GP seemed to have higher access to the emergency room, but this association was not statistically significant. The lack of significance may be explained considering the specific population assessed, generally healthy with a low rate of access to the emergency room.

## 4. Conclusions

In conclusion, less than half of the sample knew about the HD and the majority of the students did not use it. Besides, the knowledge about the HD was associated with a different use of healthcare services. Other information about the use of healthcare resources was highlighted; e.g., a high percentage of students consulted a private specialist and almost three out of four assumed medications without consulting a physician regularly. Notably, the medications without medical prescriptions more frequently used were NSAIDs and antibiotics. Therefore, our results showed which issues must be addressed in planning and designing information and education campaigns targeting university students with the aim of raising awareness about HD and a more appropriate use of healthcare services and medications. Moreover, the data of the present paper suggest that further studies are required to better understand the framework connected with the university students' access to PHC.

## Figures and Tables

**Table 1:** Having the general practitioner far away: chi-square, Fisher's exact, and Mann–Whitney *U*-tests.

Characteristics		Having the general practitioner (GP) far away		Missing	*p* value
		No (*n* = 237)	Yes (*n* = 151)		
Gender	Female	150 (63.29)	97 (64.67)	0	0.784^*∗*^
	Male	87 (36.71)	53 (35.33)		
Nationality	Italian	227 (95.78)	149 (98.68)	0	0.138^*∗∗*^
	Other	10 (4.22)	3 (1.32)		
Age		23 (22–25)	23 (22–25)	0	0.990^*∗∗∗*^
Year of university		3 (2–4)	2 (1–3)	0	<0.001^*∗∗∗*^
Years passed from the transfer to Turin		3 (2–5)	2 (1–4)	6	0.011^*∗∗∗*^
Degree of course	Bachelor's degree	103 (44.02)	65 (43.92)	6	<0.001^*∗*^
	Master's degree (second-level qualification)	39 (16.67)^b^	51 (34.46)^a^		
	Master's degree (single cycle)	92 (39.32)^a^	32 (21.62)^b^		
Kind of course	Medicine and related courses	65 (27.43)^a^	22 (14.57)^b^	0	0.020^*∗*^
	Natural Sciences	48 (20.25)	42 (27.81)		
	Legal, Political, Economic, or Social Sciences	35 (14.77)	18 (11.92)		
	Humanities	86 (36.29)	68 (45.03)		
	Agricultural or Veterinary	3 (1.27)	1 (0.66)		
Having gone to a doctor privately^#^	Yes	151 (63.71)	83 (54.97)	0	0.086^*∗*^
	No	86 (36.295)	68 (45.03)		
Number of visits to doctor on call^#^		0 (0–0)	0 (0–0)	0	0.611^*∗∗∗*^
Number of visits to GP^#^		3 (2–8)	2 (0–5)	0	<0.001^*∗∗∗*^
Number of visits to emergency room^#^		0 (0–1)	0 (0–1)	0	0.615^*∗∗∗*^
Chronic disease	Yes	26 (10.97)	15 (9.93)	0	0.746^*∗*^
	No	211 (89.03)	136 (90.07)		
Followed by a specialist regularly	Yes	107 (45.15)	72 (47.68)	0	0.625^*∗*^
	No	130 (54.85)	79 (52.32)		
Taking medications regularly	Yes	83 (35.02)	47 (31.13)	0	0.428^*∗*^
	No	154 (64.98)	104 (68.87)		
Using medications without medical prescription	Yes	170 (71.73)	112 (74.17)	0	0.599^*∗*^
	No	67 (28.27)	39 (25.83)		
Knowing about the Healthcare Domicile	Yes	95 (40.08)	81 (53.64)	0	0.009^*∗*^
	No	142 (59.92)	70 (46.36)		

Figures are median (IQR) or number (%). ^*∗*^Obtained via chi-square test. ^*∗∗*^Obtained via Fisher's exact test. ^*∗∗∗*^Obtained via Mann–Whitney *U*-test. ^a^Adjusted residual >1.96. ^b^Adjusted residual < −1.96. ^#^Since the beginning of the university.

**Table 2:** Logistic regression analyses about having the general practitioner far away and knowing about Healthcare Domicile.

Characteristics		Having the GP far away						Knowing about Healthcare Domicile					
		Univariate			Multivariable^*∗*^			Univariate			Multivariable^*∗∗*^		
		OR	95% CI	*p*	Adj OR	95% CI	*p*	OR	95% CI	*p*	Adj OR	95% CI	*p*
Female		1.06	0.69–1.63	0.784	0.76	0.22–2.63	0.659	0.85	0.56–1.28	0.433	1.08	0.68–1.71	0.755
Italian		3.28	0.71–15.19	0.128	18.51	1.14–300.28	0.040	0.83	0.26–2.61	0.743			
Age		0.98	0.91–1.05	0.518	0.86	0.62–1.19	0.364	1.16	1.07–1.25	0.001	1.13	1.02–1.26	0.024
Year of university		0.73	0.63–0.85	0.001	0.70	0.41–1.19	0.192	1.13	0.99–1.28	0.059	0.99	0.81–1.23	0.968
Years passed from the transfer to Turin		0.79	0.66–0.94	0.009	1.18	0.82–1.69	0.379	1.04	0.89–1.21	0.646			
Degree of course	Bachelor's degree	Ref.	—	—	Ref.	—	—	Ref.	—	—	Ref.	—	—
	Master's degree (second-level qualification)	2.07	1.23–3.48	0.006	1.47	0.27–7.90	0.651	1.89	1.13–3.18	0.016	1.46	0.78–2.75	0.245
	Master's degree (single cycle)	0.55	0.33–0.92	0.022	2.27	0.20–25.69	0.507	1.44	0.90–2.30	0.129	0.73	0.33–1.63	0.447
Kind of course	Medicine and related courses	Ref.	—	—	Ref.	—	—	Ref.	—	—	Ref.	—	—
	Natural Sciences	2.59	1.37–4.89	0.003	6.74	0.62–73.57	0.118	0.57	0.31–1.03	0.062	0.42	0.18–1.01	0.052
	Legal, Political, Economic, or Social Sciences	1.52	0.72–3.20	0.272	2.22	0.17–29.32	0.544	0.43	0.21–0.88	0.020	0.34	0.14–0.86	0.023
	Humanities	2.34	1.31–4.17	0.004	36.70	2.39–564.53	0.010	0.61	0.36–1.04	0.070	0.41	0.16–1.05	0.062
	Agricultural or Veterinary	0.98	0.10–9.96	0.990	Empty			0.78	0.10–5.76	0.804	0.63	0.07–6.17	0.695
Having gone to a doctor privately^#^		0.70	0.46–1.05	0.087	0.30	0.08–1.06	0.062	0.67	0.45–1.01	0.057	0.58	0.37–0.91	0.019
Number of visits to doctor on call^#^		0.97	0.83–1.15	0.756				1.33	1.08–1.65	0.008	1.37	1.09–1.74	0.008
Number of visits to GP^#^		0.96	0.92–0.99	0.014	0.97	0.90–1.05	0.466	1.01	0.99–1.03	0.354			
Number of visits to emergency room^#^		1.05	0.91–1.22	0.479				1.05	0.91–1.22	0.487			
Chronic disease		0.90	0.46–1.75	0.746				1.17	0.61–2.23	0.642			
Followed by a specialist regularly		1.11	0.74–1.67	0.625				0.99	0.66–1.48	0.968			
Taking medications regularly		0.84	0.54–1.30	0.428				0.96	0.63–1.46	0.834			
Using medications without medical prescription		1.13	0.71–1.80	0.599				0.60	0.38–0.93	0.024	0.59	0.36–0.97	0.036
Knowing about the Healthcare Domicile		1.73	1.15–2.61	0.009	0.08	0.02–0.33	0.001						

^#^Since the beginning of the university. ^*∗*^Adjusted for gender, nationality, age, year of university, years passed from the transfer to Turin, degree of course, kind of course, having gone to a doctor privately, number of visits to GP, and knowing about the Healthcare Domicile. ^*∗∗*^Adjusted for gender, age, year of university, degree of the course, kind of the course, having gone to a doctor privately, number of visits to the doctor on call, and using medications without medical prescription.

**Table 3:** Not moving the GP even if aware of Healthcare Domicile: chi-square, Fisher's, and Mann–Whitney *U*-tests.

Characteristics		Not moving the GP even if aware about Healthcare Domicile		Missing	*p* value
		No (*n* = 23)	Yes (*n* = 80)		
Gender	Female	16 (69.57)	54 (68.35)	0	0.912 ^*∗*^
	Male	7 (30.43)	25 (31.65)		
Nationality	Italian	23 (100)	79 (98.75)	0	1.000^*∗∗*^
	Other	0 (0)	1 (1.25)		
Age		25 (22–27)	24 (22–26)	0	0.158 ^*∗∗∗*^
Year of university		3 (2–6)	2 (1–3)	0	0.003^*∗∗∗*^
Years passed from the transfer to Turin		2 (2–5)	2 (1–4)	3	0.222 ^*∗∗∗*^
Degree of course	Bachelor's degree	8 (34.78)	31 (39.24)	0	0.120 ^*∗*^
	Master's degree (second-level qualification)	5 (21.74)	30 (37.97)		
	Master's degree (single cycle)	10 (43.48)	18 (22.78)		
Kind of course	Medicine and related courses	9 (39.13)^a^	12 (15.00)^b^	0	0.017 ^*∗*^
	Natural Sciences	6 (26.09)	21 (26.25)		
	Legal, Political, Economic, or Social Sciences	4 (17.39)	8 (10.00)		
	Humanities	4 (17.39)^b^	39 (48.75)^a^		
	Agricultural or Veterinary	Empty	Empty		
Having gone to a doctor privately^#^	Yes	17 (73.91)	36 (45.00)	0	0.014^*∗*^
	No	6 (26.09)	44 (55.00)		
Number of visits to doctor on call^#^		0 (0–0.5)	1 (0–2)	0	0.003^*∗∗∗*^
Number of visits to GP^#^		2 (0–6)	5 (2–10)	0	0.031^*∗∗∗*^
Number of visits to emergency room^#^		0 (0–1)	1 (0–2)	0	0.140^*∗∗∗*^
Chronic disease	Yes	4 (17.39)	7 (8.75)	0	0.259^*∗∗*^
	No	19 (82.61)	73 (91.25)		
Followed by a specialist regularly	Yes	10 (43.48)	39 (48.75)	0	0.655^*∗*^
	No	13 (56.52)	41 (51.25)		
Taking medications regularly	Yes	9 (39.13)	26 (32.50)	0	0.554^*∗*^
	No	14 (60.87)	54 (67.50)		
Using medications without medical prescription	Yes	16 (69.57)	56 (70.00)	0	0.968^*∗*^
	No	7 (30.43)	24 (30.00)		
	No	142 (59.92)	24 (30.00)		

Figures are median (IQR) or number (%). ^*∗*^Obtained via chi-square test. ^*∗∗*^Obtained via Fisher's exact test. ^*∗∗∗*^Obtained via Mann–Whitney *U*-test. ^a^Adjusted residual >1.96. ^b^Adjusted residual < −1.96. ^#^ Since the beginning of the university.

**Table 4:** Logistic regression analyses about not moving the GP even if aware of Healthcare Domicile.

Characteristics		Not moving the GP even if aware of Healthcare Domicile					
		Univariate			Multivariable^*∗*^		
		OR	95% CI	*p*	Adj OR	95% CI	*p*
Female		0.95	0.35–2.59	0.912	1.20	0.26–5.49	0.815
Age		0.89	0.74–1.06	0.183	0.78	0.51–1.15	0.201
Year of university		0.58	0.42–0.79	0.001	0.42	0.17–1.02	0.054
Years passed from the transfer to Turin		0.87	0.69–1.09	0.228	2.46	1.13–5.34	0.023
Degree of course	Bachelor's degree	Ref.	—	—	Ref.	—	—
	Master's degree (second-level qualification)	1.55	0.45–5.27	0.484	5.25	0.52–52.61	0.158
	Master's degree (single cycle)	0.46	0.16–1.39	0.170	2.90	0.20–42.49	0.436
Kind of course	Medicine and related courses	Ref.	—	—	Ref.	—	—
	Natural Sciences	2.63	0.75–9.19	0.131	2.04	0.14–30.06	0.604
	Legal, Political, Economic, or Social Sciences	1.5	0.34–6.58	0.591	0.77	0.031–18.88	0.873
	Humanities	7.31	1.91–28.03	0.004	36.18	1.31–997.56	0.034
	Agricultural or Veterinary	Empty			Empty		
Having gone to a doctor privately^#^		0.29	0.10–0.81	0.018	0.16	0.03–0.82	0.028
Number of visits to doctor on call^#^		0.64	0.44–0.93	0.018	0.40	0.20–0.78	0.007
Number of visits to GP^#^		0.97	0.91–1.03	0.279			
Number of visits to emergency room^#^		0.89	0.67–1.20	0.452			
Chronic disease		0.46	0.12–1.72	0.246	0.17	0.02–1.59	0.119
Followed by a specialist regularly		1.24	0.49–3.15	0.656			
Taking medications regularly		0.75	0.29–1.95	0.555			
Using medications without medical prescription		1.02	0.37–2.80	0.968			

#Since the beginning of the university. ^*∗*^Adjusted for gender, age, year of university, years passed from the transfer to Turin, degree of the course, kind of the course, having gone to a doctor privately, number of visits to the doctor on call, and chronic disease.

## Data Availability

The data used to support the findings of this study are included within the article.
